# Supplementary material for: Evaluation of the Safety and Immunogenicity of Fractional Intradermal COVID-19 Vaccines as a Booster: A Pilot Study
Source: Vaccines (Basel). 2022 Sep 8;10(9):1497. doi: 10.3390/vaccines10091497 (PMC9505744; doi:10.3390/vaccines10091497)
Supplement: Supplementary file 1 [file vaccines-10-01497-s001.zip › vaccines-1856464-supplementary.pdf]

Table S1. Baseline characteristics of the subjects of each group in initial phase.

Primary series (IM) - Booster (ID)		All	CoronaVac - BNT162b2	CoronaVac - ChAdOx1	ChAdOx1 - BNT162b2	ChAdOx1 - ChAdOx1	BNT162b2 - BNT162b2	BNT162b2 - ChAdOx1	*p*-value
		**Types of Vaccines**							
**Initial phase**									
Number of subjects	n (%)	58 (100.0)	10 (17.2)	10 (17.2)	10 (17.2)	10 (17.2)	9 (15.5)	9 (15.5)	-
Age (years)	Median (IQR)	43.0 (35.0, 48.0)	40.5 (28.0, 45.0)	42.0 (24.0, 44.0)	49.0 (46.0, 51.0)	45.0 (43.0, 50.0)	41.0 (36.0, 43.0)	39.0 (35.0, 43.0)	0.323
Male	n (%)	19 (32.8)	3 (15.8)	3 (15.8)	2 (10.5)	1 (5.3)	7 (36.8)	3 (15.8)	0.055
Body mass index: BMI (kg/m^2)	Median (IQR)	23.4 (20.6, 25.9)	25.6 (24.4, 27.5)	20.0 (18.0, 23.2)	24.7 (22.3, 25.9)	22.4 (20.8, 27.1)	24.1 (20.7, 24.5)	22.6 (20.9, 23.6)	0.181
Interval between last dose of primary series and booster (weeks)	Median (IQR)	9.1 (6.0, 10.0)	9.0 (8.1, 10.0)	9.3 (9.1, 10.0)	6.0 (6.0, 6.0)	6.0 (6.0, 6.0)	10.0 (10.0, 10.0)	10.0 (10.0, 10.0)	0.463

Table S2. Immunogenicity response following intradermal booster vaccination by types of vaccines in primary series (2-dose intramuscular) and booster (ID) in the initial phase.

Primary series (IM) - Booster (ID)	All	CoronaVac - BNT162b2	CoronaVac - ChAdOx1	ChAdOx1 - BNT162b2	ChAdOx1 - ChAdOx1	BNT162b2 - BNT162b2	BNT162b2 - ChAdOx1	*p*-value
		**Types of Vaccines**						
**Number of subjects**	**(n=58)**	**n=10**	**n=10**	**n=10**	**n=10**	**n=9**	**n=9**	
**anti-RBD IgG (BAU/mL)**								
GMC at pre-booster vaccination (95% CI)	218.66 (168.09, 284.44)	94.87 (51.88, 173.49)	112.93 (65.94, 193.39)	188.60 (119.77, 296.97)	240.80 (126.88, 457.00)	353.21 (200.27, 622.96)	755.29 (479.91, 1188.69)	<0.001*
p-value		0.632		0.491		0.028*		
GMC at post-booster vaccination (95% CI)	1,188.24 (889.78, 1586.81)	3,209.64 (1974.92, 5216.32)	2,810.93 (1850.26, 4270.39)	1,489.54 (797.55, 2781.92)	309.48 (179.16, 534.62)	848.58 (413.62, 1740.94)	734.93 (437.25, 1235.29)	<0.001*
p-value		0.645		<0.001*		0.713		
GMR between post-booster vaccination /pre-booster vaccination (95% CI)	5.43 (3.66, 8.06)	33.83 (19.34, 59.16)	24.89 (14.14, 43.81)	7.90 (5.80, 10.76)	1.29 (1.10, 1.50)	2.40 (1.52, 3.81)	0.97 (0.80, 1.19)	<0.001*
p-value		0.394		<0.001*		<0.001*		
**Live virus plaque reduction neutralization titers (PRNT$_{50}$) post intradermal boosting**								
GMT against delta strain at post-booster vaccination (95% CI)	200.64 (154.07, 261.29)	409.40 (257.48, 650.96)	431.83 (258.86, 720.36)	300.78 (184.08, 491.46)	95.60 (58.82, 155.37)	116.52 (52.38, 259.21)	96.99 (48.31, 194.72)	<0.001*
p-value		0.863		0.002*		0.695		
GMT against omicron strain at post-booster vaccination (95% CI)	27.52 (14.57, 51.97)	76.34 (39.57, 147.29)	130.21 (72.30, 234.51)	51.75 (24.89, 107.57)	6.71 (1.29, 34.74)	53.31 (26.87, 105.78)	1.94 (0.09, 43.11)	<0.001*
p-value		0.188		0.019*		0.029*		
Number (%) with PRNT$_{50}$ against omicron strain ≤1:10	6 (10.34)	-	-	-	3 (30.00)	-	3 (33.33)	0.017*

* p ≤ 0.05. One-way ANOVA and unpaired t-test with parametric assumptions satisfied was determined *P*-value among those who received any vaccinations. Abbreviation: BAU/mL: binding antibody unit/mL, GMC: geometric mean concentration, GMT: geometric mean titer, GM: geometric mean, GMR: geometric mean ratio.

Table S3. Immunogenicity response following intradermal booster vaccination by types of vaccines in primary series (2-dose intramuscular) and booster (ID) in the extended phase.

Primary series (IM) - Booster (ID)	All	CoronaVac - BNT162b2	CoronaVac - ChAdOx1	ChAdOx1 - BNT162b2	*p*-value
	Types of Vaccines				
**Number of subjects**	**(n=135)**	**n=45**	**n=45**	**n=45**	
**anti-RBD IgG (BAU/mL)**					
GMC at pre-booster vaccination (95% CI)	54.13 (42.99, 68.15)	34.26 (23.76, 49.39)	27.52 (18.71, 40.46)	168.21 (137.42, 205.90)	<0.001*
GMC at post-booster vaccination (95% CI)	1,494.31 (1308.69, 1706.27)	1,978.48 (1595.09, 2454.02)	1,326.85 (1025.61, 1716.57)	1,271.08 (1032.54, 1564.71)	0.011
GMR between post-booster vaccination /pre-booster vaccination (95% CI)	27.61 (21.98, 34.68)	57.75 (42.53, 78.42)	48.22 (33.40, 69.62)	7.56 (6.34, 9.00)	<0.001*
**Live virus plaque reduction neutralization titers ($PRNT_{50}$) post intradermal boosting**					
GMT against delta strain at post-booster vaccination (95% CI)	257.20 (221.85, 298.18)	287.62 (225.33, 367.14)	206.15 (149.94, 283.41)	286.95 (235.14, 350.19)	0.112
GMT against omicron strain at post-booster vaccination (95% CI)	45.52 (38.24, 54.19)	52.74 (38.10, 72.99)	45.82 (32.98, 63.66)	39.05 (29.96, 50.89)	0.382
GMR: delta/omicron at post-booster vaccination (95%CI)	5.65 (4.89, 6.52)	5.45 (4.11, 7.24)	4.50 (3.53, 5.73)	7.35 (5.94, 9.10)	0.020*
Number (%) with $PRNT_{50}$ against omicron strain ≤1:10	19 (9.63)	6 (13.33)	5 (11.11)	2 (4.44)	0.331
**Number of subjects**	**(n=60)**	**n=20**	**n=20**	**n=20**	
**ELISpot responses (SFU/10^6 cells)**					
ELISpot-S GM at pre-booster vaccination (95%CI)	11.01 (6.80, 17.81)	4.22 (2.07, 8.61)	5.89 (2.79, 12.41)	53.68 (28.15, 102.37)	<0.001*
ELISpot-NMO GM at pre-booster vaccination (95%CI)	8.31 (5.39, 12.82)	9.41 (4.37, 20.27)	20.15 (9.98, 40.68)	3.03 (1.60, 5.75)	<0.001*
ELISpot-S GM at post-booster vaccination (95% CI)	105.43 (71.88, 154.64)	129.75 (62.58, 269.02)	46.82 (25.16, 87.14)	192.93 (108.44, 343.22)	0.006*
ELISpot-NMO GM at post-booster vaccination (95% CI)	25.24 (15.19, 41.93)	45.97 (18.02, 117.28)	21.35 (10.88, 41.88)	16.38 (5.58, 48.14)	0.229
ELISpot-S GMR: post-booster vaccination /pre-booster vaccination (95% CI)	9.58 (6.15, 14.91)	30.77 (12.69, 74.62)	7.95 (4.22, 14.97)	3.59 (2.14, 6.03)	<0.001*
ELISpot-NMO GMR: post-booster vaccination /pre-booster vaccination (95% CI)	3.04 (1.84, 5.02)	4.88 (1.98, 12.04)	1.06 (0.67, 1.67)	5.41 (18.4, 15.89)	0.010*

* $p \leq 0.05$. One-way ANOVA and was determined *p*-value among those who received any vaccinations. Abbreviation: BAU/mL: binding antibody unit/mL, GMC: geometric mean concentration, GMT: geometric mean titer, GM: geometric mean, GMR: geometric mean ratio, SFU/10^6 cells: spot forming unit per million cells.

Table S4. Adverse events following intradermal COVID-19 vaccination in the booster in the extended phase.

Adverse events	Type of vaccines				*p*-value
**Primary series (IM) - Booster (ID)**	**All**	**CoronaVac - BNT162b2**	**CoronaVac - ChAdOx1**	**ChAdOx1 - BNT162b2**	
**Number of subjects**	**n=135**	**n=45**	**n=45**	**n=45**	
**Injection site reaction, n (%)**	122 (90.37)	40 (88.89)	41 (91.11)	41 (91.11)	
Mild, n (%)	98 (72.59)	31 (68.89)	29 (64.44)	38 (84.44)	0.148
Moderate, n (%)	24 (17.78	9 (20.00)	12 (26.67)	3 (6.67)	
**Any Systemic reaction, n (%)**	107 (79.26)	37 (82.22)	39 (86.67)	31 (68.88)	
Mild, n (%)	92 (68.15)	33 (73.33)	30 (66.67)	29 (64.44)	0.056
Moderate, n (%)	15 (11.11)	4 (8.89)	9 (20.00)	2 (4.44)	
**Myalgia, n (%)**	69 (51.11)	24 (53.33)	24 (53.33)	21 (46.67)	
Mild, n (%)	60 (44.44)	21 (46.67)	22 (48.89)	17 (37.78)	0.745
Moderate, n (%)	8 (5.93)	2 (4.44)	2 (4.44)	4 (8.89)	
**Fatigue, n (%)**	57 (42.22)	20 (44.44)	22 (48.89)	15 (33.33)	
Mild, n (%)	57 (42.22)	20 (44.44)	22 (48.89)	15 (33.33)	0.306
Moderate, n (%)	0 (0.00)	0 (0.00)	0 (0.00)	0 (0.00)	
**Headache, n (%)**	63 (46.67)	19 (42.22)	26 (57.78)	18 (40.00)	
Mild, n (%)	50 (37.04)	15 (33.33)	18 (40.00)	17 (37.78)	0.109
Moderate, n (%)	13 (9.63)	4 (8.89)	8 (17.78)	1 (2.22)	
**Fever, n (%)**	3 (2.22)	1 (2.22)	1 (2.22)	1 (2.22)	
Mild, n (%)	1 (0.74)	1 (2.22)	0 (0.00)	0 (0.00)	0.365
Moderate, n (%)	2 (1.48)	0 (0.00)	1 (2.22)	1 (2.22)	
**Flu-like, n (%)**	1 (0.74)	0 (0.00)	0 (0.00)	1 (2.22)	
Mild, n (%)	1 (0.74)	0 (0.00)	0 (0.00)	1 (2.22)	0.365
Moderate, n (%)	0 (0.00)	0 (0.00)	0 (0.00)	0 (0.00)	
**Somnolence**, n (%)	7 (5.19)	3 (6.67)	3 (6.67)	1 (2.22)	
Mild, n (%)	7 (5.19)	3 (6.67)	3 (6.67)	1 (2.22)	0.547
Moderate, n (%)	0 (0.00)	0 (0.00)	0 (0.00)	0 (0.00)	
**Diarrhea, n (%)**	8 (5.93)	4 (8.89)	3 (6.67)	1 (2.22)	
Mild, n (%)	8 (5.93)	4 (8.89)	3 (6.67)	1 (2.22)	0.395
Moderate, n (%)	0 (0.00)	0 (0.00)	0 (0.00)	0 (0.00)	
**Nausea, n (%)**	15 (11.11)	5 (11.11)	8 (17.78)	2 (4.44)	
Mild, n (%)	15 (11.11)	5 (11.11)	8 (17.78)	2 (4.44)	0.132
Moderate, n (%)	0 (0.00)	0 (0.00)	0 (0.00)	0 (0.00)	
**Vomit, n (%)**	1 (0.74)	1 (2.22)	0 (0.00)	0 (0.00)	
Mild, n (%)	1 (0.74)	1 (2.22)	0 (0.00)	0 (0.00)	0.365
Moderate, n (%)	0 (0.00)	0 (0.00)	0 (0.00)	0 (0.00)	

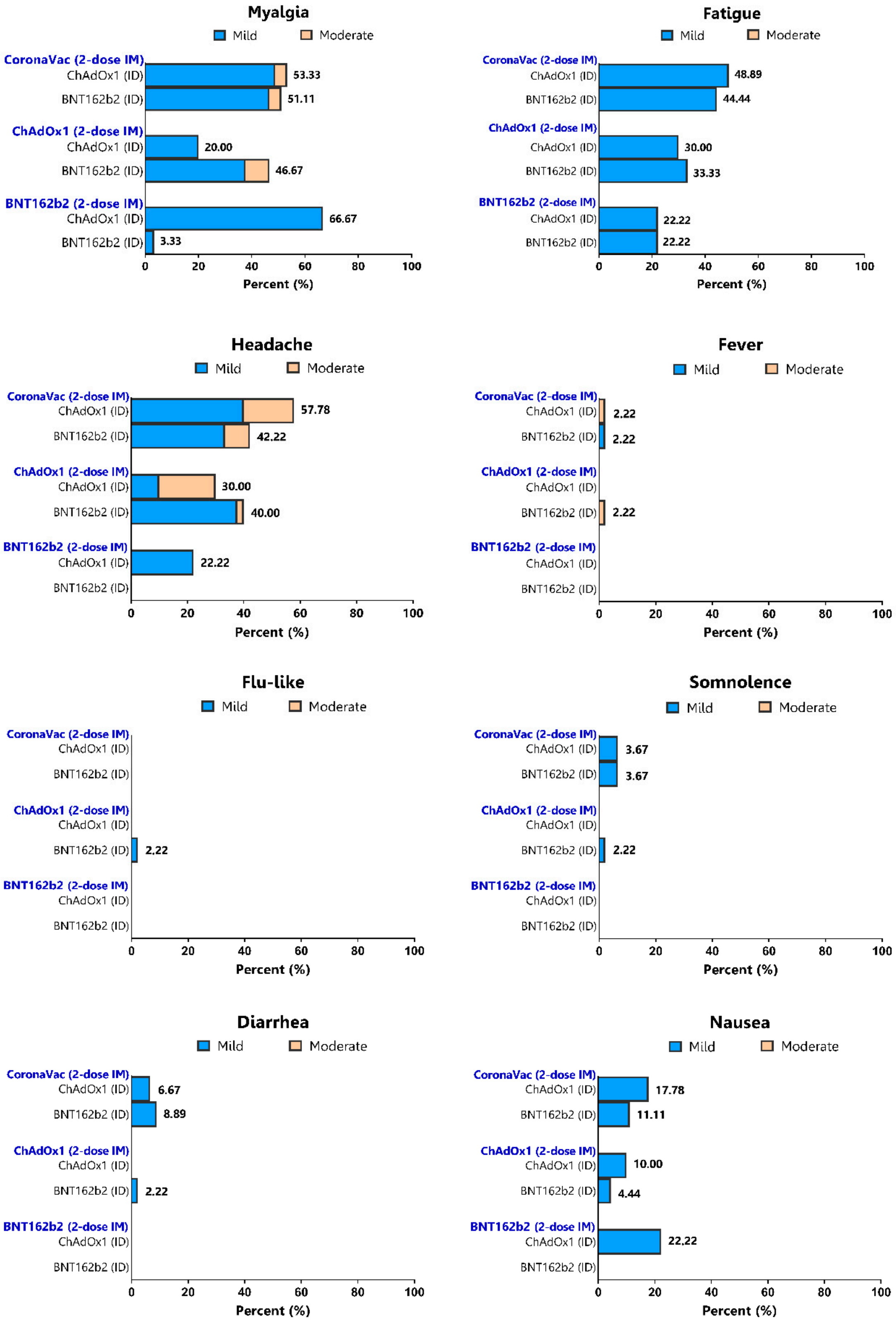

Figure S1. Adverse events by self-report following intradermal boosting.
